# Near-Infrared Transillumination for Occlusal Carious Lesion Detection: A Retrospective Reliability Study

**DOI:** 10.3390/diagnostics13010036

**Published:** 2022-12-23

**Authors:** Muna Mohamed Nur, Lydia Vazquez, Clara Anton Y. Otero, Caroline Giacobino, Ivo Krejci, Marwa Abdelaziz

**Affiliations:** 1 Division of Cariology and Endodontology, University Clinics of Dental Medicine (CUMD), University of Geneva, rue Michel-Servet 1, 1211 Geneva, Switzerland; 2 Dental Radiology, Department of Orofacial Rehabilitation, University Clinics of Dental Medicine (CUMD), University of Geneva, 1205 Geneva, Switzerland; 3 Clinical Research Center, University Hospitals of Geneva, 1205 Geneva, Switzerland

**Keywords:** caries, occlusal caries detection, near-infrared transillumination (NIRT), DIAGNOcam, bitewing radiograph, ICDAS, monitoring, occlusal carious lesions, early occlusal lesions

## Abstract

The aim of this study was to assess the reliability of three diagnostic methods (near-infrared transillumination (NIRT), bitewing radiographs (BW), and clinical images (CI)) to detect occlusal carious lesions in a low caries risk population. This retrospective analysis included one hundred and eighty-eight occlusal surfaces, scored as sound surface, early lesion, or distinct lesion. We evaluated the agreement between and within the methods over time. Kappa statistics tested the correlation between the methods. Examiners detected occlusal early lesions more frequently with visual examination and NIRT and the same lesions were confirmed on the 2-year follow-up. Within the limitations of this study, we were able to establish that early occlusal lesions can be detected and monitored over time using NIRT and visual exam, while BW scores showed mostly sound surfaces at both examinations. NIRT combined with clinical examination can be considered appropriate to detect and monitor early enamel caries on the occlusal surface in low caries-risk populations.

## 1. Introduction

Although dental caries is one of the most common chronic diseases worldwide, the prevalence, extent, and severity of dental caries have declined significantly over the past decades in most populations [1]. The development of a carious lesion is a dynamic process that can be arrested if diagnosed before the symptoms appear. Early caries diagnosis is essential in daily practice as it helps detection long before symptoms appear which can allow the control of the carious process through prevention or early intervention. The search for a better method of caries detection in different stages is still a subject of constant evolution. Ideally, a diagnostic device should allow the detection of all stages of the carious process, including early and non-cavitated carious lesions [2]. Existing diagnostic methods have been found to be insufficient in detecting early lesions and the need for additional methods has long been acknowledged [3].

The task becomes more challenging when it comes to occlusal surfaces. This is due to the accumulation of debris and plaque that are difficult to remove because of the complex morphology of the pits and fissures. It is also assumed that the occlusal lesion is initiated on the fissure walls and is therefore concealed by superimposed sound tissue [4,5]. In addition, the extensive use of fluoride causes superficial remineralization of enamel delaying the cavitation process while dentine caries remain hidden bellow [6]. This leads to the development of lesions that could only be perceptible radiographically and are known as “hidden caries” or lesions that are even more challenging to detect if they are not yet visible radiographically. The latter are termed “questionable” since they are suspected only by their appearance such as the presence of surface opacities, staining, or roughness [7].

The most common and conventional method for occlusal caries detection is the visual-tactile method; however, it is not sensitive enough for early lesions [8]. The International Caries Detection and Assessment System (ICDAS) was established by an international group of researchers with the goal of designing an internationally recognized caries detection system that would also allow the assessment of caries activity [9]. The ICDAS classification criteria and associated estimates of caries activity is based on the histological extension of lesions spreading into the tooth tissues [9,10]. ICDAS presented good reproducibility and accuracy in detecting occlusal carious lesions in the outer half of the enamel in permanent teeth, [11] and in various stages of the disease process [12].

Bitewing radiographs (BW) are usually complementary to the visual-tactile method. These X-rays are, however, not reliable or sensitive enough to detect early non–cavitated occlusal caries due to overlapping topography [13]. When an occlusal carious lesion is detected on a BW, the lesion may have already reached the middle third of dentine, well beyond remineralization interventions [14,15,16].

The use of near-infrared transillumination (NIRT) for early carious lesions detection has been described for more than a decade [14,17]. The enamel appears highly transparent when illuminated using NIRT and dentine appears darker than enamel because it scatters light more strongly. During the caries process, micropores are formed in the lesion due to the partial dissolution of the individual mineral crystals. These small pores behave as scattering centers, strongly scattering visible and near infrared light. Carious lesions appear dark because they scatter and absorb light more than surrounding healthy tissue [17,18,19]. Real-time images are displayed on a monitor and can be stored to allow monitoring. [20,21] The area and contrast of occlusal lesions in NIRT images can be correlated with lesion severity, and lesions that penetrate into dentine have significantly higher contrast than those in the enamel [18,22]. Several studies have shown NIRT to have higher sensitivity than BW to detect both proximal and occlusal carious lesion [23,24,25,26,27]. NIRT has also shown higher inter-examiner and intra-examiner reliability than BW [22,28,29]. The device DIAGNOcam (Kavo, Biberach, Germany) used in this study is the first marketed device that uses NIRT technology and has been tested in multiple studies [21,30,31,32].

The aim of this study was to assess the reliability of three diagnostic methods NIRT, BW, and visual examination using ICDAS criteria based on photographs, to detect and monitor occlusal carious lesions in a low caries risk population.

## 2. Materials and Methods

### 2.1. Materials

Dental records of eighteen dental students in the final year were examined and collected after obtaining their oral consent for the anonymous use of the data.

BW radiographs had been taken by different practitioners with an interval of two years (+/−3 months) (exposure parameters were 70 kV, 7 mA, 0.1620130.20 s). Several digital imaging plates size 2 and CS 7600 scanning system (Carestream Health, Rochester, NY, USA) were used.

DIAGNOcam 2170U (Kavo, Biberach, Germany) and the reusable tip for adults were used to acquire the NIRT images, with the KaVo integrated desktop (KID) software V 2.4.2.

Intraoral photographs were taken with a digital camera (Nikon D5300, Nikkor 105 micro).

### 2.2. Data Collection

Inclusion criteria: last year dental students (18 years and older), with available bitewing radiographs with around 2 years intervals (3 months less or more were tolerated), and the NIRT images of both years and the clinical images of the first examination were included.

Exclusion criteria: students with incomplete data (missing X-rays, photographs or NIRT images).

The primary analysis excluded six subjects from eighteen due to incomplete or missing data in the digital records. Thus, this retrospective study collected data from twelve subjects whose ages varied from 22 to 32 years old.

Clinical intraoral photographs were available for the first examination only. BW and NIRT images were available from the initial examination and the two years follow-up assessment.

A PowerPoint file was prepared for each subject. The clinical photographs, BW and NIRT images were displayed for each quadrant on a slide (i.e., four slides per subject). One slide was lacking for one subject because the follow-up NIRT assessment was missing. After removing the patient’s initials from the slides, the forty-seven slides were mixed, randomly reorganized, then numbered granting complete and irreversible anonymity. Each of the final forty-seven slides with the clinical images, BW, and NIRT images of both examinations were then redistributed to six slides: Visual 1, BW 1, BW 2, NIRT1, NIRT 2, and all data combined. The final data set included 188 occlusal surfaces that were assessed and scored. Due to the retrospective study design, no sample size calculation was possible prior to the study.

### 2.3. Training and Data Interpretation

#### 2.3.1. Examiners

Examiner 1 (MA): has been working with NIRT and teaching and researching new diagnostic methods at the university of Geneva since 2012.

Examiner 2 (LV): is the head of the radiology department and has been working and teaching at the university for over 20 years.

#### 2.3.2. Training

Examiners followed the ICDAS e-learning program, developed by the ICDAS Foundation which is a 90-minute course used to introduce the criteria to new users. The ICDAS e-learning program has been shown to improve the implementation of the diagnostic skills of students for the detection of occlusal caries lesions [33].

#### 2.3.3. Data Scoring and Interpretation

Each diagnostic method was scored independently. For NIRT and BW each year was also scored separately following the scoring system shown in Table 1 [34]. Each score was recorded on an excel file for final analysis. The scoring was done again after one month from the first analysis using the same procedure described above to determine intra-examiner agreement for sixty random surfaces.

Recently published Standard reporting of caries detection and diagnostic studies (STARCARDDS) were followed whenever applicable [35].

### 2.4. Data Analysis

A total of 188 occlusal surfaces were scored twice based on the following diagnostic techniques: BW, NIRT, and Visual evaluation of clinical images. The Visual method was used only for the first assessment.

Possible scores were: 0, 1, 2, 3, 4 as well as “Missing”, “Not interpretable” and “Filled”. When measuring inter and intra-reliability, the teeth whose score was “Missing”, “Not interpretable”, or “Filled” were excluded. A score of 0 was recorded as “Sound”, a score of 1 was recorded as “Early” and a score of 2, 3, and 4 were recorded as “Distinct”. The choice to combine codes 2, 3 and 4 was made because codes 3 and 4 were rare within the pool of cases we have (young low-risk caries adults) and most scores were 1 and 2.

Differences in kappa coefficients between both examinations were reported for the NIRT and BW methods. In addition, differences in kappa coefficients between each pair of diagnostic techniques for the first examination and between NIRT and BW for the follow-up examination were reported. This was done for each diagnostic method and each examination with Cohen’s kappa coefficient, which assumes the score is a categorical variable. It is bounded by one. A value of one implies perfect agreement and a negative value indicates that agreement was less than would be expected just by chance [36].

Intra-examiner reliability (the reliability of measurements of the initial assessment and the follow-up assessment by a given examiner with a fixed diagnostic method) was measured. This was done for each diagnostic method with Cohen’s kappa coefficient. Differences in kappa coefficients between NIRT and BW were reported.

Landis and Koch (1977) [36] classified values as follows: <0 as indicating no agreement, 0–0.20 as slight, 0.21–0.40 as fair, 0.41–0.60 as moderate, 0.61–0.80 as substantial, and 0.81–1 as almost perfect agreement.

All kappa coefficients and differences were reported along their 95% confidence interval. The confidence intervals of the kappa coefficients were based upon the variance estimates, whereas those for the difference were obtained by the bootstrap. Bootstrapping is any test or metric that uses random sampling with replacement (e.g., mimicking the sampling process) and falls under the broader class of resampling methods.

The *p* values for testing the equality of two kappa coefficients were obtained by a permutation test. Permutation tests work by resampling the observed data many times in order to determine a p-value for the test.

Statistical analyses were performed using R (R Foundation for Statistical Computing, Vienna, Austria) with the package “psych” for the computation of kappa coefficients.

## 3. Results

This retrospective study analyzed data from twelve subjects whose ages varied between 22 and 32 years old, a total of 188 occlusal surfaces were scored.

### 3.1. Frequency Distribution of Scores for Each Diagnostic Method

Table 2 provides an overview of the frequency (%) distribution of all scored surfaces by the experienced examiners including missing, not interpretable, and filled surfaces. The clinical images showed that 53 (28.2%) of the surfaces were restored (filled).

Table 3 illustrate the frequency distribution of the scores for each diagnostic method (Clinical images (CI), near-infrared transillumination (NIRT), and bitewing radiographs (BW) for both assessments. It can be observed that for the BW method, “Sound” is the most frequent rating, whereas for NIRT and Visual, “Early” is the most frequent one. The BW showed that only 3% of surfaces examined has occlusal carious lesions.

### 3.2. Agreement between and within Methods

The inter-rater reliability test for the clinicians done on 60 surfaces was substantial to excellent (between 0.61–0.85) depending on the method.

Measures of agreement between methods and within methods based on the scoring were assessed with Cohen’s kappa coefficients (Table 4). The coefficients showed no agreement between BW and NIRT, moderate agreement for BW, and substantial agreement for NIRT in the two examinations.

The results from this study show a low correlation between BW and visual which further confirms that early occlusal lesions cannot be detected by BW. Kappa values were higher for CI vs. NIRT (0.3) compared to CI vs. BW (0.03–0.04) in the current study confirming that NIR is a superior method to bitewings to detect early enamel caries on the occlusal surface.

Table 5 below shows the distribution of scores of surfaces with full data (no missing data), from the experienced examiners for the 3 methods on both assessments, we can clearly see the agreement within methods (BW1 vs. BW2, NIRT 1 vs. NIRT2)**.**

## 4. Discussion

In the present clinical study, ICDAS scores of occlusal surfaces based on clinical photographs were compared to scores based on NIRT images and digital intra-oral radiographs. The results show that more early occlusal lesions were detected using NIRT followed by clinical images, while BW scores showed mostly sound surfaces at both examinations (first and second assessments) as shown in Table 3.

A similar in vivo study [37] that compared the three methods found that most carious lesions were detected using visual examination followed by NIRT and then BWs. Our finding might be explained by the stricter criteria we used for NIRT images. We considered any visible changes in the occlusal fissure system as an early sign of demineralization based on some of our unpublished in vitro work on extracted teeth with occlusal lesions.

Another reason might be the fact that we used photographs for the visual examination. Advanced technologies have made the use of intraoral photographs in clinical examination simpler, easier, and relatively cheap. Clinical images allow archiving, remote scoring, scoring from multiple examiners, and longitudinal analysis [38]. They could be especially useful in identifying occlusal carious lesions. It has been reported that the assessment of clinical images as a method of detection of occlusal dentine caries had higher sensitivity than visual examination using histology as a reference standard [38]. Clinical images also showed good inter-and intra-examiner reliability that was similar to visual examination [38,39,40].

The low percentage of carious lesions detected on BWs in our study corroborates the finding of previous studies concerning occlusal carious lesion detection on radiographs. When occlusal lesions are detected on radiographs they usually have reached the middle third of the dentine [41]. Previous studies have shown that BWs have negligible diagnostic value to detect enamel carious lesions and occlusal superficial dentine carious lesions [42,43]. NIRT could therefore be a good adjunct to clinical examination for detecting early occlusal caries. Several studies have suggested that combining the visual examination with another method could improve the accuracy of occlusal carious lesions detection [44,45]. These studies also confirmed the findings of our study.

The population of this study is considered at very low risk for caries development, the students in dental medicine are well-informed about hygiene and they rarely develop new caries. This was confirmed by the lack of lesion progression observed between the two assessments two years apart.

The results discussed above confirm that occlusal carious lesions detection using NIR combined with visual assessment is more reliable than X-ray.

A previous study has shown that NIRT lesion detection is closest to clinical results in occlusal carious lesions and superior to other detection methods like dyes, laser fluorescence, and X-rays. Thus, NIRT would be the most useful method as an adjunct to visual inspection [26].

The results from this study show a low agreement between BW and visual examination which further confirms previous studies reporting that early occlusal carious lesions cannot be detected by BW. A limited number of studies compared NIRT with the visual method on the occlusal surface [25,26]. One study showed a very high correlation between clinical examination and NIRT with a kappa value of 0.99 [25], while the other study found that NIRT also correlated the most with visual examination with r = 0.51 [26]. The reason why Lara-Capi et al. [25] showed high concordance between visual and NIRT methods could be the simpler scoring system used. All dark occlusal shadows were scored as 1 While in our study we used a comprehensive scoring system from 0–4 where scores 1 and 2 indicated thin and wide grey shadows in the enamel, respectively, and scores 3 and 4 indicated shadows less and more than 2 mm into the dentine. Without a gold standard reference, the only statement that can be reported for comparing NIRT to visual examination is that NIRT was able to detect more occlusal lesions.

Further development of the monitoring concept using NIRT images is required to enable more precise follow-up of occlusal and proximal lesions. Some reports showed possible longitudinal monitoring for occlusal and proximal carious lesions using NIRT [23,32]. Although our study did not look into the management of early occlusal caries, we must emphasize the slow progression of these lesions in this sample of a low caries risk population and how this impacts their management. Our NIRT and BW scores showed that 90% of scored lesions remained stable in a 2-year period indicating a good possibility of their arrest or reversal. This is a valid argument for conservative management of early and questionable occlusal carious lesions. To our knowledge, not many studies discussed the characteristics and progression rate of these types of lesions, and a consensus is required on how to properly monitor and manage them [7]. One study following up on questionable occlusal carious lesions for twenty months found that 90% of lesions at the end of the period only required monitoring and no invasive treatment was required [7].

The advantages of NIRT over BW are that NIRT can overcome overlapping of the enamel, is non-ionizing, and can indicate the relative position of the lesion [29]. It can also analyze images in real-time. NIRT is most useful for early detection and patient follow-up and monitoring [23]. However, to monitor lesions using NIRT image, it is necessary to obtain comparable images at each recall. Detection and monitoring require further development and automatization. Advances in using artificial intelligence to detect and monitor carious lesions on X-rays and NIRT images seem to be promising [46].

There are still many questions that need to be answered as criteria for when NIRT vs. BW is needed. Examiners should make that decision based on many factors. In the meantime, NIRT can reduce the use of BW [47]. Other studies have confirmed our conclusion that that NIRT actually can be a valid alternative, especially for incipient lesions on the occlusal surface [22].

The design of the study does not allow histological validation of the observed carious lesion as it is a retrospective study. Enamel carious lesions cannot be validated in vivo studies because they are managed through preventive measures, unlike dentinal carious lesions, which could be validated when they are restored. In vitro studies can validate lesions via histological methods. Many studies recognized the problem of in vivo validation of enamel carious lesions [20,26,47]. The issue of gold standards and how to validate carious in vivo are not novel and have been recognized since the early 1990s [6]. To overcome these limitations, a study conducted validation of carious lesions by performing both in vivo detection in planned extraction of third molar teeth and then performing histological examination after extraction. This study showed NIRT to have the best correlation with histology and the highest sensitivity and accuracy rates to detect early occlusal carious lesions [48]. Kuhnisch et al. have also stated the difficulty of creating an appropriate in vitro setup because the optical properties of the different embedding materials are not equivalent to the periodontal tissue or anatomy.

The population of this study was a small low caries-risk and highly dental hygiene-conscious group. This can be considered a limitation as the results may not be fully generalizable to the whole population.

Sensitivity and specificity values were not calculated in this retrospective study due to the problem stated above. Accurate sensitivity and specificity values cannot be obtained when novel methods like NIRT are compared to imperfect “gold standards” such as the visual method and BW. However, several prior studies showed NIRT higher sensitivity than BW to detect occlusal caries [22,27]. This does not exclude the possibility of more false positives with NIRT imaging, however, when suspected and before taking any therapeutical measures, it is possible to differentiate lesion from discoloration by cleaning the occlusal surface with sandblasting to remove any discoloration. The truth remains that even in case of a false positive diagnosis for an early lesion, the treatment would be increasing the preventive measures or sealing the surface if the patient is at risk. These minimally invasive interventions are harmless and painless and would only provide extra protection for that surface. It must be stressed that using new technologies such as NIRT while practicing old-fashioned dentistry where any lesion detected required a restoration can be problematic and harmful to the patients and is considered as over-treatment. Such tools require a modern caries management approach based on risk assessment, close monitoring, and the use of minimal invasive therapeutical restorations such as sealing and infiltration of early lesions [49,50,51].

It would have been interesting to have the clinical images for a visual evaluation for the second assessment too and to ensure a standardized way for taking the images with properly dried surfaces, but as a drawback of a retrospective study, only the use of available data is possible.

A strength of this study is the monitoring of the same lesions longitudinally so the second reading could be regarded as a validation method for the first reading. NIRT scores showed that over 90% of scores remained the same, signifying slow or no change amongst the low-risk sample in this study and indicating that NIRT could be a useful tool for monitoring and personalized caries management.

Further research on a population with higher caries risk can further confirm the monitoring potential; in our low caries risk population, the monitoring served more as a confirmation and validation for the lesion presented.

## 5. Conclusions

Within the limitations of this study, we were able to establish that more early occlusal carious lesions were observed using NIRT and visual exam, while BW scores showed mostly sound surfaces at both examinations with two years interval. NIRT can be considered appropriate to detect and monitor initial enamel carious lesions on the occlusal surface in low caries risk populations. This method has the benefit of being X-ray free and non-invasive and can be reused as necessary.

## Figures and Tables

**Table 1:** Scoring System of the three detection methods used in this study (adapted from Gomez et al. 2015 [34]). Visual evaluation using ICDAS based on clinical images (CI), near-infrared transillumination (NIRT), and bitewing radiographs (BW) were scored into 5 categories as described above. Score 1 was considered as early lesions while scores 2, 3 and 4 were considered as distinct lesions.

Score	Visual	NIRT	BW
0	Sound 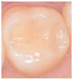	No shadow 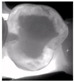	No radiolucency 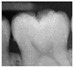
1 Early	First Visual change in enamel 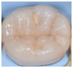	Thin grey shadow into enamel 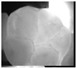	Radiolucency in outer half of enamel 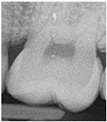
2 Distinct	Distinct Visual change in enamel 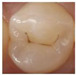	Wide grey shadow into enamel 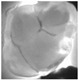	Radiolucency in inner half if enamel +/- Enamel dentin junction 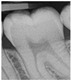
3 Distinct	Localized enamel breakdown 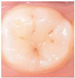	Shadow less than 2mm in dentine 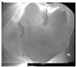	Radiolucency limited to outer 1/3 of dentine 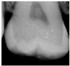
4 Distinct	Underlying dentinal shadow 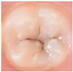	Shadow more than 2 mm in dentine 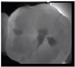	Radiolucency passed 1/3 of dentine 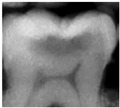

**Table 2:** Frequency (%) distribution of scores given by the experienced dentists for each diagnostic method for both assessments, (Clinical images (CI), near-infrared transillumination (NIRT), and bitewing radiographs (BW)), including missing, filled, and non-interpretable surfaces. The most relevant results are in bold.

	NIRT1	NIRT2	BW1	BW2	CI1
**#**	188	188	188	188	188
Sound	10 (5.3)	10 (5.4)	**132 (70.2)**	**131 (69.7)**	48 (25.5)
Early	**83 (44.1)**	**78 (41.9)**	1 (0.5)	2 (1.1)	**54 (28.7)**
Distinct	31 (16.5)	26 (14)	4 (2.1)	3 (1.6)	30 (16)
Missing	12 (6.4)	21 (11.3)	3 (1.6)	3 (1.6)	3 (1.6)
Not interpretable	3 (1.6)	3 (1.6)	1 (0.5)	0	0
Filled	49 (26.1)	48 (25.8)	47 (25)	49 (26.1)	53 (28.2)
NA (empty cell)	0	2	0	0	0

**Table 3:** Frequency (%) distribution of scores for each diagnostic method for both assessments (Clinical images (CI), near-infrared transillumination (NIRT), and bitewing radiographs (BW)) excluding missing, filled, and non-interpretable surfaces. The most relevant results are in bold.

	NIRT 1	NIRT 2	BW 1	BW 2	CI 1
#	124	114	137	136	132
Sound	10 (8.1%)	10 (8.8%)	**132 (96.4%)**	**131 (96.3%)**	48 (36.4%)
Early	**83 (66.9%)**	**78 (68.4%)**	1 (0.7%)	2 (1.5%)	**54 (40.9%)**
Distinct	31 (25%)	26 (22.8%)	4 (2.9%)	3 (2.2%)	30 (22.7%)

**Table 4:** Cohen’s kappa coefficients with 95% confidence intervals, between methods, and within methods. (Near-infrared transillumination (NIRT), and bitewing radiographs (BW)). The most relevant results are in bold. Surfaces with one missing, filled, non-interpretable image at any point were excluded.

	Kappa (N = 109)
BW1 versus NIRT 1	0.02 (−0.03 to 0.07)
BW2 versus NIRT 2	0.02 (−0.02 to 0.06)
BW 1 versus BW 2	**0.52 (0.32 to 0.72)**
NIRT 1 versus NIRT 2	**0.72 (0.56 to 0.88)**

**Table 5:** Cross-tabulations for 109 occlusal surfaces with full data of experienced examiners (Clinical images (CI), near-infrared transillumination (NIRT), and bitewing radiographs (BW)). The agreement of both BW assessments is mostly on sound surfaces while NIRT assessments are on early caries. While most of the early lesions detected on clinical images (CI) were scored sound on BW, NIRT was able to detect more early lesions than CI. The most relevant results are in bold.

**BW 1**	**BW 2**			**NIRT 1**	**NIRT 2**		
	Sound	Early	Distinct		Sound	Early	Distinct
**Sound**	**79(72.5%)**	5(4.6%)	1(0.9%)	Sound	4 (3.7%)	2 (1.8%)	0
**Early**	11(10.1%)	**12(11%)**	0	Early	2 (1.8%)	**83 (76.1%)**	1(0.9%)
**Distinct**	0	0	1 (0.9%)	Distinct	0	5(4.6%)	12(11%)
**BW 1**	**CI**			**NIRT 1**	**CI**		
	Sound	Early	Distinct		Sound	Early	Distinct
**Sound**	**32 (29.4%)**	**42 (38.5%)**	11 (10.1%)	Sound	6 (5.5%)	0	0
**Early**	5 (4.6%)	10 (9.2%)	8 (7.3%)	Early	**27 (24.8%)**	**49 (45%)**	10 (9.2%)
**Distinct**	0	0	1 (0.9%)	Distinct	4 (3.7%)	3 (2.8%)	10 (9.2%)

## Data Availability

Data can be provided upon request. Please contact the corresponding author.
